# The burden of varicella from a parent's perspective and its societal impact in The Netherlands: an Internet survey

**DOI:** 10.1186/1471-2334-11-320

**Published:** 2011-11-17

**Authors:** Judith H Wolleswinkel-van den Bosch, Anouk M Speets, Hans C Rümke, Pearl D Gumbs, Sander C Fortanier

**Affiliations:** 1 Pallas, health research and consultancy BV, Rotterdam, the Netherlands; 2 Vaxinostics BV, Rotterdam, the Netherlands; 3 GlaxoSmithKline, Zeist, the Netherlands

## Abstract

**Background:**

Varicella is a common childhood disease. Only 5% of first varicella-zoster-virus infections occur asymptomatically. Most data on the burden of varicella stem from health service databases. This study aims to provide insight in the burden of varicella from a parent's perspective including cases outside the healthcare system.

**Methods:**

An internet questionnaire was developed for parents in the Netherlands to report health care resource use and productivity losses during the varicella episode in their child younger than 6 years. 11,367 invitations were sent out to members with children of an internet panel of a market research agency. 4,168 (37%) parents started the questionnaire (response rate), of which 360 (9%) stopped before completion and 1,838 (44%) were out of the target group. In total 1,970 parents completed the questionnaire. The questionnaire provided a symptom list ranging from common symptoms, such as skin vesicles, itching to fits or convulsions. A posteriori, in the analyses, the symptoms 'skin infections', 'fits/convulsions', 'unconsciousness', and 'balance and movement disorders' were labelled as complications. There was no restriction to time since the varicella episode for inclusion in the analyses.

**Results:**

The 1,970 respondents had in total 2,899 children aged younger than six years, of which 2,564 (88%) children had had varicella. In 62% of the episodes the parent did not seek medical help. In 18% of all episodes symptoms labelled as complications were reported; in 11% of all episodes parents visited a medical doctor (MD) for a complication. Reporting of complications did not differ (X^2 ^; p = 0.964) between children with a recent (≤ 12 months ago) or a more distant (> 12 months) history of varicella. Prescription drugs were used in 12% of the children with varicella; OTC drugs in 72%. Parents reported work loss in 17% of the varicella-episodes (23% when MD visit; 14% when no MD-visit) for on average 14 hours, which equals to 2.5 hours of work loss for any given varicella-episode.

**Conclusions:**

This study shows the full spectrum of varicella-episodes and associated healthcare use, including the large proportion of cases not seeking medical care and the societal impact associated with those cases.

## Background

Varicella is a common childhood disease. The seropositivity increases sharply with age from 18% for both 0- and 1-year-olds, to 49%, 59%, 76% and 93% for 2-, 3-, 4- and 5-year-olds, respectively [1]. Only about 5% of first varicella-zoster-virus (VZV) infections occur asymptomatically [2]. The contagious period lasts from 1-2 days before up to 5-7 days after appearance of the rash [3]. Varicella usually is an uncomplicated, self-limiting disease. However, serious complications leading to hospitalisation and, sometimes, death do occur. In the Netherlands, the general practitioner (GP) consultation rate (all ages) for varicella is 253 per 100,000 populations leading to an estimated 40,000 visits to the GP. The hospital admission rate is 1.3 per 100,000 leading to 200 hospital admissions, and there are about two deaths each year on a population of 16 million [1].

Universal vaccination for varicella has been introduced in several developed countries (e.g. Germany, Australia, Japan, Canada and the United States), showing dramatic decline of disease incidence in the latter [4]. In the Netherlands the decision to include varicella in the national immunisation vaccination programme is pending. The main points of uncertainty relate to the impact of varicella vaccination on the incidence of zoster infections later in life [5], and uncertainty related to the burden of complications following varicella infection, and the indirect non-medical costs associated with varicella [2]. Most data we have on the burden of varicella stem from hospital data and GP-databases. However, the Netherlands is known for more conservative health seeking behaviour as compared to other European countries. The GP contact rate is only lower in Finland and Greece [6].

Hence we will miss a considerable burden of varicella in the population when we look at health service based data, because not all of the parents will seek medical help. We acknowledge that those cases can be expected to be mild, but they still might have an impact on society through work loss by the parents.

The aim of this study is to provide insight in the burden of varicella from a parent's perspective. We will describe the frequency of symptoms, including complications, the use of medical resources, and the societal impact including cases, which do not enter the healthcare system.

## Methods

### Questionnaire and Study Population

We developed a questionnaire retrospectively asking parents about symptoms, health care use and productivity losses related to the most recent episode experienced of varicella of one of their children younger than six years. The domains covered by the questionnaire included background information on the parent and family (e.g. age, education, number of children in the family) characteristics of the disease episode (symptoms and complications, duration, frequency), the use of medical resources (number of physician and emergency room (ER) visits, telephone consultations, hospitalisations, prescription and over-the-counter (OTC) drug use including zero use; diagnostic tests and surgical interventions were not included), and productivity loss by the caregivers (work days lost, loss of productivity at work, loss of leisure time). The questionnaire was sent electronically to a sample of parents with young children from the Netherlands, who participated in an Internet access panel of Survey Sampling International (SSI), a market research agency in Rotterdam, the Netherlands. Children under six years were included in the analyses, matching the age-specific incidence of chickenpox in the Netherlands. The panel, which consists of people who have elected to participate in surveys sent out by SSI on behalf of its clients, is a general consumer panel, not specifically set up for health-related subjects. There was no financial incentive specifically related to this internet survey, but SSI donates a small amount to charity for every complete questionnaire returned, and there is a prize draw with small prizes for the participants. It was technically impossible for respondents to complete the questionnaire more than once. In total 11,367 invitations were sent out by SSI to panel members with children, of which 4,168 (37%) started the questionnaire (the response rate). Of the people who started the questionnaire 360 (9%) stopped and 1,838 (44%) were out of the target group. In total 1,970 parents completed the questionnaire. SSI collects information about their panel members such as family composition, including age of the children. However this information is not always complete (e.g. SSI only knows the panel member has children, but does not know the age) or can be out dated. The questionnaire contained questions to check if the respondent was eligible for the study. If not, they were screened out.

The questionnaire included questions on education and geographical distribution. The demographics of the sample showed that the geographical distribution of the respondents was representative of the population in the Netherlands. The indicator of socio-economic status (education) was slightly higher in this survey (low education 15%, medium 50%, high 35% versus low 29%, medium 46% and high 25% in the Dutch population (all differences being significant due to large numbers for the Dutch population). The mean number of children of the respondents (1.5 children) was comparable with the Dutch population (1.7 children).

Parents were asked to provide details about the most recent varicella episode of their young child (under six years of age). When they had more than one child under the age of six, they were asked to complete the questionnaire for all children. All data were self-reported by the parents.

There was no restriction to time since the varicella episode to be included in the results. Thus the varicella episode could have been little less than six years prior to the questionnaire at a maximum. However, questions related to productivity loss were only presented to parents with children who had varicella up to and including 12 months ago. As a consequence, data on work absenteeism and productivity loss, and leisure time loss were based on a subsample of 904 children with a recent (i.e. ≤12 months) history of varicella. Questions on the frequency and type of symptoms, including complications, were addressed to the whole sample. The questionnaire was field tested by a small group (n = 10) of parents to identify possible gaps and unclear phrasing of the questions. The parents received a test link in order to complete the questionnaire online. They were subsequently interviewed about the feasibility of the questionnaire. The Internet survey was sent out and completed in June 2008.

The questionnaire provided a symptom list ranging from common symptoms of varicella, such as skin vesicles, itching, fever, troubled sleeping, and skin infection to fits or convulsions. Parents were asked to indicate whether their child had one or more of the symptoms listed and whether they had visited a medical doctor for one of these symptoms. Parents were asked to report all the health care services used related to the varicella episode including physician visits, ER visits, hospitalisations, prescription drugs, and OTC drugs (diagnostic tests and surgical interventions were not included). A posteriori, in the analyses, we labelled 'skin infections', 'fits/convulsions', 'unconsciousness', and 'balance and movement disorders' as complications.

### Indirect Resource Use (Productivity Losses)

Productivity losses included hours of absence from a paid or unpaid job, as well as loss of productivity at work and loss of hours of leisure time. Absence from an unpaid job was defined as not being able to (1) provide usual informal care to others; (2) carry out any other voluntary work; or (3) carry out activities around the house [7]. To investigate loss of productivity at work, we asked whether the parents felt less productive during work because of their child's illness (e.g., because they could not concentrate properly). If so, parents with a paid job were asked to estimate how many extra hours they would have needed to be as productive as when their child was not ill. This question has shown to yield conservative results for loss of productivity at work [8]. Loss of leisure time was defined as any time during free weekdays or weekends that was spent at a physician or pharmacy visit because of the child's illness. The parent who filled out the questionnaire was asked to fill out any productivity loss hours for their partner, the child's grandparents, or other informal caregivers as well. For each type of productivity loss hours (absence from work, loss of productivity at work, and loss of leisure time), the hours lost are presented for all informal caregivers combined (i.e., the total hours missed due to the disease episode).

The questionnaire was developed by the authors of this paper and programmed by SSI in close collaboration with the authors. In developing the questionnaire, both the Productivity and Disease Questionnaire (PRODISQ) [9] and the Health and Labour Questionnaire (HLQ) [10] were studied, and relevant items on productivity losses were included in the survey questionnaire. We did not use PRODISQ or HLQ because these questionnaires were developed to measure productivity loss due to health problems in the working adults and contain many items not relevant to our study.

### Statistical analyses

The data were mainly categorical. The data-analysis was descriptive. Results were addressed in terms of absolute numbers, percentages, means, standard deviations, medians, 95% confidence intervals and ranges. Chi-square analyses and Mann-Whitney tests were used to test for statistical differences between groups when the data allowed such comparisons. Data were analysed using SPSS for Windows version 14.0.

## Results

### Study population and symptoms

The response rate of the questionnaire was 37%. Data were available for 1,970 respondents (mean age 35.5 years; 82% female respondents) with in total 2,899 children aged younger than six years, of which 2,564 (88%) children reported a history of varicella. Table 1 provides information on the characteristics of the children with a history of varicella. In 35% (n = 904) of cases the time elapsed between the disease episode and filling out the questionnaire was shorter than or equalled 12 months. The mean age of these children (2.7 years (SD 1.5) was younger than for those who had had varicella over one year ago (4.8 years SD 1.0). The most frequent symptoms of varicella were skin vesicles (98%), itching (92%), listlessness (91%) and fever (78%). In 472 children (18%) one or more symptoms that can be seen as complications were reported by the parents; 411 children (16%) with skin infection or inflammation, 115 children (5%) with balance or movement disorders, 85 children (3%) with fits/convulsions and 65 children (3%) with unconsciousness (Table 2).

**Table 1:** Characteristics of children < 6 years of age with history of varicella, the Netherlands, 2007

	N (%)
Total with history of varicella	2,564 (100)
Gender; girls	1,236 (48.2)
Mean age ± SD in years (range)	4.0 ± 1.3 years (range 0.2-5.9)
Age groups (in years)	
0	55 (2.1)
1	198 (7.7)
2	341 (13.3)
3	521 (20.3)
4	704 (27.5)
5	745 (29.1)

Time elapsed at survey since varicella episode	

Currently ill	55 (2.1)
Last month	88 (3.4)
2-5 months ago	331 (12.9)
6-12 months ago	430 (16.8)
1-2 years ago	719 (28.0)
More than 2 years ago	941 (36.7)
	
Mean age ± SD in years (range) history of varicella ≤ 12 months (n = 904)	2.7 ± 1.5 years (range 0.2-5.9)

**Table 2:** Symptoms during an episode of varicella* in children < 6 years of age, the Netherlands, 2007

	No medical doctor's visit (n = 1,591)	Medical doctor's visit (n = 973)	0-5 years (n = 2,564)
	**N (%)**	**N (%)**	**N (%)**
	
**Symptoms**			
Skin vesicles^¥^	1,552 (97.5)	964 (99.1)	2,516 (98.1)
Itching^‡^	1,452 (91.3)	905 (93.0)	2,357 (91.9)
Listlessness^¶^	1,417 (89.1)	908 (93.3)	2,325 (90.7)
Fever^¶^	1,167 (73.4)	830 (85.3)	1,997 (77.9)
Troubled sleeping^¶^	941 (59.1)	706 (72.6)	1,647 (64.2)
Troubled eating or drinking	785 (49.3)	619 (63.6)	1,404 (54.8)
Cold/coughing^¶^	647 (40.7)	551 (56.6)	1,198 (46.7)
Mouth vesicles^¶^	574 (36.1)	457 (47.0)	1,031 (40.2)
Skin haemorrhage^¶^	530 (33.3)	439 (45.1)	969 (37.8)
Diarrhoea^¶^	390 (24.5)	421 (43.3)	811 (31.6)
Vomiting^¶^	210 (13.2)	261 (26.8)	471 (18.4)
Skin infection or inflammation^¶^	168 (10.6)	243 (25.0)	411 (16.0)
Balance or movement disorder^¶^	37 (2.3)	78 (8.0)	115 (4.5)
Fits/convulsions^¶^	31 (1.9)	54 (5.5)	85 (3.3)
Unconsciousness^†^	27 (1.7)	38 (3.9)	65 (2.5)
			
Other symptoms^¶ ^§	8 (0.5)	37 (3.8)	45 (1.8)

* More than one symptom or complication could be filled out; ¶ Chi-square test, p = 0.000; † Chi-square test, p = 0.001;

¥ Chi-square test, p = 0.006; ‡ Chi-square test, p = 0.115; § Broad range of complaints (e.g. asthma, otitis, laryngitis, conjunctivitis, cystitis, dehydration)

In 973 children with an episode of varicella the parents sought medical care (Table 2). When we only take into account the complications for which the parents visited a medical doctor there were 278 children (11% of total reported episodes) with complications; 10% skin infections, 3% balance or movement disorders, 2% fits/convulsions and 2% unconsciousness.

As expected, all symptoms, except for itching, were significantly more present in children who visited a medical doctor. Symptoms that can be seen as complications, such as skin infections, fits/convulsions, balance and movement disorders, and unconsciousness were two to three times as present among children who visited a medical doctor than those who did not (Table 2). The prevalence of symptoms did not differ among the younger (0-3 years) and older (4-5 years) children. The rate of complications of varicella was relatively stable in one-year age groups, ranging from 18% in one-year olds to 19% in five-year olds. The complication rate in children younger than one year was lower with 7%. The reported complication rate did not statistically significant differ (Chi-square test; p = 0.964) between children with a recent history (≤ 12 months ago) of varicella and those who had varicella more than one year ago (e.g. skin infection respectively 16% versus 16%; convulsions 3% versus 4%).

The mean duration of an episode of varicella was 8.4 days (SD 4.5 days) and was significantly higher in children who consulted any medical doctor (9.3 days versus 7.8 days) (Mann-Whitney test; p = 0.000).

### Health care use

Only 38% (n = 973) of the parents consulted a physician when their child was ill with varicella. The percentage physician consultations was lower among children without complications (33%; n = 686) than with complications (61%; n = 287) (Chi-square test; p < 0.05). In most cases the consultation was a face-to-face consultation with a GP during weekdays and/or a telephone consultation (Table 3). There was no difference in the proportion visiting a medical doctor between the age groups 0-3 years and 4-5 years. According to the parents, the occurrence of skin vesicles was the most important reason to consult a GP or specialist care (43%), followed by itching (20%) and fever (19%) (results not shown).

**Table 3:** Health care use during an episode of varicella in children < 6 years of age, the Netherlands, 2007

	Children without complications (n = 2,092)		Children with one or more complications (n = 472)		All children (n = 2,564)	
	**N (%)**	**Mean number of** **consultations (range)**	**N (%)**	**Mean number of** **consultations (range)**	**N (%)**	**Mean number of** **consultations (range)**

GP telephone consultation	324 (15.5)	1.1 (1-3)	119 (25.2)	1.2 (1-4)	443 (17.3)	1.1 (1-4)
GP visit (not during weekend/night)	344 (16.4)	1.1 (1-5)	179 (37.9)	1.2 (1-10)	543 (21.2)	1.1 (1-10)
GP visited during weekend	22 (1.1)	1.0 (1-2)	27 (5.7)	1.2 (1-5)	49 (1.9)	1.1 (1-5)
GP visit during night	4 (0.2)	1.0 (1-1)	4 (0.8)	1.0 (1-1)	8 (0.3)	1.0 (1-1)
GP home visit	3 (0.1)	1.3 (1-2)	12 (2.5)	1.6 (1-3)	15 (0.6)	1.5 (1-3)
Visit emergency department	8 (0.4)	1.1 (1-2)	25 (5.3)	1.2 (1-3)	33 (1.3)	1.2 (1-3)
Consultation specialist care	6 (0.3)	1.2 (1-2)	14 (3.0)	1.6 (1-5)	20 (0.8)	1.5 (1-5)
						
No medical doctor's consultation	1406 (67.2)		185 (39.2)		1,591 (62.1)	
Medical doctor's consultation	686 (32.8)		287 (60.8)		973 (37.9)	
						
Hospitalizations	3 (0.1)	Mean duration: 4.7 days	14 (3.0)	Mean duration: 7.1 days	17 (0.7)	Mean duration 6.7 days
						
Drugs prescribed	161 (7.7)		149 (31.6)		310 (12.1)	
Antibiotics	55 (2.6)		82 (17.4)		137 (5.3)	
Other drugs	106 (5.1)		67 (14.2)		173 (6.7)	
OTC drugs	1501 (71.7)		355 (75.2)		1,856 (72.4)	

All differences statistically significant (Chi-square test, p < 0.05)

Sixty-one per cent (n = 288) of the cases with one or more complications visited a medical doctor and, 32% received a prescription drug (Table 3). This pattern was largely similar for all complications separately as well. The medical visit rate was highest among children with balance and movement disorders, 68%.

Seventeen children (1%) were hospitalized for the symptoms and/or complications of varicella, for a mean duration of 6.7 days (SD 5.6; range 1-21 days). As expected, the hospitalization rate was significantly higher in children with complications (overall 3%; 9% in cases with unconsciousness and 5% in cases with convulsions). Children who were hospitalized had combinations of different symptoms and/or complications of varicella. The most important symptoms leading to hospitalization were fever (47%), unconsciousness (35%), skin infections (29%) and fits/convulsions (24%). Seven children were hospitalized due to frequently reported complications of varicella such as asthma, pneumonia, and otitis of both ears [11].

Use of prescription drugs was reported in 12% of the children with varicella (n = 310), being considerably higher in children with complications (Table 3). In 72% of all children with an episode of varicella OTC drugs were bought. In that case there was no difference between children with or without complications. Parents who visited a medical doctor bought slightly more often OTC drugs for their child with varicella (76%) compared with parents who did not visit a medical doctor (70%).

### Productivity loss

Of the 2,564 children with varicella, 1,749 children attended school or any form of day care including home care or staying with family members. Varicella caused absence from school or day care in 57% of the children (65% in children visiting a medical doctor; 52% in those who did not) who visited school or any form of day care. The mean duration of the absence was 4.1 days (SD 3.5 days) and the absence was significant longer in children who consulted a medical doctor (mean 4.8 days versus 3.5 days) (Mann-Whitney test; p = 0.000).

Parents reported time lost from a paid job in 17% of the varicella-episodes. When hours were lost, this was on average 14.3 hours (hours of all caregivers combined). Caregivers who consulted a GP or specialist care took more often days off from their paid job than caregivers who did not consult a medical doctor (23% versus 14%). Their absence duration was also higher; on average 17.3 hours versus 11.1 hours (Chi-square test; p = 0.000) (Table 4).

**Table 4:** Key results on societal impact of varicella in children < 6 years of age in the Netherlands, 2007.

	No consultation of medical doctor (n = 550)	Consultation of medical doctor (n = 354)	Total (n = 904)
**Paid job**			
Number of children for which caregiver(s) had to stay home n (%)	76 (13.8)	80 (22.6)	156 (17.3)
Mean absence ± SD in hours (range)*	11.1 ± 7.9 (1-40)	17.3 ± 14.3 (1-60)	14.3 ± 12.0 (1-60)
**Unpaid job**			
Number of children for which caregiver(s) had to stay home n (%)	31 (5.6)	20 (5.6)	51 (5.6)
Mean absence ± SD in hours (range)*	17.8 ± 20.2 (1-90)	22.1 ± 26.1 (1-100)	19.2 ± 22.2 (1-100)

Productivity loss at work**			

Number of parents reporting productivity loss n (%)	34 (6.2)	39 (11.0)	73 (8.1)
Mean productivity loss ± SD in hours (range)*	7.8 ± 9.3 (1-40)	6.7 ± 6.0 (1-30)	7.2 ± 7.7 (1-40)

Leisure time loss***			

Number of parents reporting leisure time loss n (%)	151 (27.5)	198 (55.9)	349 (38.6)
Mean leisure time loss ± SD in hours (range)*	1.5 ± 6.0 (0.1-72)	2.0 ± 3.5 (0.2-24)	1.8 ± 4.7 (0.1-72)

* In the cases with absence from paid or unpaid work, productivity loss at work or leisure time loss

** For example, due to lack of sleep because of caring for the sick child

*** Leisure time lost due to doctor or pharmacy visit

Because in the majority of cases no absence from work was reported, the average work loss per varicella-episode (regardless of parental work loss involved) equals to 2.5 hours of work lost (4 hours for episodes with and 1.6 hours for episodes without consultation of a medical doctor). The main reason why parents did not need to take hours off from their paid job was the ability to switch work days with free week days.

Leave of absence from an unpaid job was reported less often but the hours taken off were longer.

We also asked parents about impaired productivity at work, due to lack of sleep or worry about their child's illness. Parents who visited a doctor reported more frequently a loss of productivity at work than parents that did not consult a medical doctor for their child with varicella (11% versus 6%) (Chi-square test; p = 0.009). However, the average amount of hours lost was significantly less in parents who consulted a medical doctor: 6.7 hours versus 7.8 hours (Mann-Whitney test; p = 0.01) (Table 4).

Many parents reported that some free time, about 2 hours, was spent on a physician or pharmacist visit (39%).

## Discussion

This study provides insight in the medical burden of varicella from a parent's perspective and in addition also on the impact on work absenteeism and productivity loss at work.

Only 38% of the parents visit a medical doctor due to symptom or complication. The whole range of symptoms associated with varicella was more present among children of parents who visited a medical doctor. On average there were about 4 hours of work lost per varicella episode when parents had visited a medical doctor, and about 1.6 hours when they did not. Parents report more symptoms than what we know from GP databases.

The results of this study were based on an Internet panel survey on varicella, with a 'response rate' of 37%. Although this seems rather low, it should be kept in mind that this is not a classic response rate as it depends on how well the parents with young children could be targeted by the market research agency. This depends on the background data available at the market research agency. When this background is incomplete, a larger, less targeted wave of invitations is sent out, which will result in relatively low response rates.

The mean age of the parents who filled out the questionnaire was 36 years (SD 5 years) - in line with expected ages of parents with children under six years old. This age group is very Internet-literate in the Netherlands with 86% of 25-54 year olds using the Internet at least once a week [12]. In most cases the mother was the respondent (82%), but we explicitly asked the respondent to fill out the data on productivity loss for other possible caregivers (e.g. father or grandparents) as well. The demographics of the sample showed that the geographical distribution of the respondents was representative of the population in the Netherlands. The educational level of the respondents was somewhat higher than reference data from the national statistics (highest education 35% versus 24%). The educational and income level of the participants can influence the outcome, as there might be a difference in use of medical resources between educational levels [13]. Socioeconomic status (measured as educational level and income) might also affect employment rate or type of employment, which could affect the results of absence from a paid job.

Comparison of results of an Internet access panel with a probability sample (random digit dialling (RDD)) has shown that internet responses were significantly more likely to agree with RDD responses when the question asked was about the respondent's personal health (nine times more likely than when not about their personal health) and when the question was factual (nine times more likely than non-factual) [14]. The questions in our questionnaire complied largely with these characteristics.

This Internet survey showed that 88% of the respondents' children aged younger than six years had a history of varicella. De Melker et al. reported a seroprevalence of varicella for children aged five years of 93%, including asymptomatic cases of varicella [1]. About 5% of first VZV infections occur asymptomatically and this internet survey did not include asymptomatic cases of varicella. Thus, the prevalence of symptomatic cases in our survey is comparable with the seroprevalence as reported by De Melker et al. [1].

The prevalence of symptoms reported in this study gives an overview of the prevalence of the most common varicella symptoms from a parent perspective. It shows, not surprisingly, that almost all children have skin vesicles and show itching. Fever occurs in about three quarters of the children, two thirds have problems with sleeping, and about half of the children have problems with eating or drinking. 11% of the parents reported an episode with complications for which they visited a medical doctor.

Parents are capable of reporting a history of varicella in their children. Several studies have shown high positive predictive value of 95% or more for parent reports [15,16]. The accuracy of self-reported data diminishes with time elapsed. We asked parents to report on a varicella episode of their children aged less than six years. We analysed whether there were differences in the report of complications for a history of varicella up and including 12 months and 12 months or more. There were no significant differences (Chi-square test; p = 0.964).

It is probably difficult for parents to distinguish between a more serious course of the varicella episode and complications. Therefore we just listed mild and more serious symptoms in the questionnaire (see Table 1) and we labelled 'skin infection or inflammation', 'balance or movement disorder', 'fits/convulsions', and 'unconsciousness' as complications afterwards in the analyses. Parents reported 'complications' in 18% of all varicella episodes irrespective of physician visit. We used lay-men vocabulary to ask about the more serious symptoms. As a consequence of that, within the 18%, symptoms like 'balance and movement disorder', 'fits/convulsions', and 'unconsciousness' will probably include mild presentations of these symptoms.

The varicella episodes with complications for which a medical doctor had been visited probably represent the more serious complications. This was the case in 11% of all varicella episodes (278/2564); in 6% of all varicella episodes drugs were prescribed in an episode with complications (149/2564). Those percentages fit in the range of data on complications of varicella published elsewhere. The complication rate in varicella in primary care ranges from 2% to 15% in other published data [2,17-23]. Although those other studies are primary care based and our study is population based, we believe that this comparison is justified. Health care use is much higher in other European countries in mild childhood diseases as compared with the Netherlands. In the Netherlands, for any given disease-episode in young children 47% of the parents visit a medical doctor, whereas this is 79% in Germany, 89% in Spain, 86% in France and 87% in Italy [24]. In other words, it is likely that in other countries less serious cases present at primary care as well, contrary to the Netherlands.

Another factor that should be taken into account to appreciate the results of the Internet survey is that the results are based on parent reports which might result in a higher prevalence estimate as compared with primary care based studies. GP's or paediatricians might only record what they consider the primary symptom for which parents came for a consultation, while parents have likely reported all symptoms they remembered about the disease episode. This might, for example, include relatively minor skin infections that a GP would normally not record. In addition, children are infected with varicella-zoster-virus early in life in the Netherlands (50% seroconversion by age two), as compared to for example Germany (50% seroconversion by age four). This might also affect the complication rate and explain differences in complication rate between countries.

Other factors that explain differences in complication rates among publications are the complications included e.g. dehydration from vomiting or diarrhoea, exacerbation of asthma, pneumonia, bacterial super infections, acute neurological disorders, bronchitis and otitis media, conjunctivitis and corneal infections, central nervous system injuries and stomatitis. Studies differ also in age groups included (e.g. up to 14 years or under 4 years), and whether or not immunocompromised children are included [2].

In case of hospitalizations, we asked the symptoms that led to admission of the child. Although the number of hospitalized children was low (n = 17), the distribution of symptoms seems more comparable with published data. For example, in Germany, febrile convulsions were present in 30% of the hospitalized children < 16 years in the pre-immunization era [25], and skin infections in 23% [26]. Super infections of the skin occurred in 37% of hospitalized children in a French study [27]. Neurological complications were reported in about one quarter of hospitalized cases in a French and German study [25,28].

Data on health care use of mild childhood diseases, and specifically the percentage of children not seeking medical care are scarce. Our results on health care use could be compared with a postal survey among parents of children aged 2-6 years in the Netherlands in 1998 [29]. IThe postal survey relates to all kinds of childhood diseases. In our study, parents did not seek medical help for 62% of the disease episodes, compared to 50% in the postal survey. There had only been telephone contact with a GP in 17% versus 23% of the disease episodes in respectively the Internet and postal survey. Use of OTC drugs was reported in the Internet survey for 72% and in the postal survey for 64% of the episodes. Results from another Internet survey indicated that 53% of the children with symptoms of otitis media did not consult a medical doctor in the Netherlands [24].

The hospitalization rate of 0.7% in children with varicella is comparable with hospitalization rates reported in other studies. The hospitalization rate reported by De Melker et al for children presenting with varicella at the GP was 0.4% for 5-9 year olds, 1.0% in 1-4 year olds and 2.7% in infants [1]. Banz et al. (2004) reported a hospitalization rate for cases with varicella or an associated complication of 0.8% in Germany [11]. He referred in the same article to other published data indicating that in the United States between 0.1% and 0.6% of children are admitted to hospital as a result of varicella or an associated complication, while data from Canada suggest a somewhat higher hospitalization rate for children during an acute episode of varicella, ranging between 0.2% and 1.5%, and in France about 0.5% of all varicella patients need hospital care due to severe complications [11].

To reduce recall bias, questions on societal impact (e.g. work absenteeism) were only asked about varicella episodes that had occurred up to and including 12 months ago; half of the varicella episodes had occurred up to 6 months ago. Results for work absenteeism (Table 4) are comparable with other studies. Seventeen per cent of the parents needed to take time off from work to care for their child. It has previously been estimated from a Dutch community study on costs of gastroenteritis that 15% of the parents had to miss work for a child's episode of rotavirus diarrhoea [30,31]. A review study on economic evaluations of varicella vaccination programmes has reported numbers of working days lost for a common case of varicella of 1.2 in Spain, 0.6 in France and 2.6 in Germany (adjusted for caregivers outside the labour market) [32]. Other studies have reported 0.6 to 3.7 working days missed for any uncomplicated case of varicella [11,33,34]. Results for the Netherlands (0.5 days for episodes with and 0.2 days for episodes without consultation of a medical doctor) are at the low end of this range, which can probably be explained by a high-proportion of part-time work (80% among women) in the Netherlands [35].

## Conclusions

In sum, this study provides insight in the full spectrum of varicella-episodes and associated healthcare use, which is of interest for an infectious disease that infects virtually all young children. In addition, insight in the proportion of cases not seeking medical care and the societal impact associated with those cases gives valuable information for the cost-effectiveness analyses on vaccine introduction in the Netherlands. This study shows the otherwise not visible burden of disease outside the healthcare system, which is of particular importance in countries with conservative health seeking behaviour.

## Competing interests

JHWvdB is director of Pallas, health research and consultancy BV. Pallas conducted epidemiological, observational studies for GSK in the field of vaccine preventable diseases. The author does not own any stocks, nor has other interests in this company. AMS is senior researcher at Pallas, health research and consultancy BV. Pallas conducted epidemiological, observational studies for GSK in the field of vaccine preventable diseases. AMS does not own any stocks, nor has other interests in this company. HCR was medical director of Vaxinostics BV, board member of the Dutch Vaccines Group, member of the committee for the National Immunisation Programme of the Health Council at the time of preparing the manuscript. Vaxinostics conducted clinical studies on MMVR vaccines for GSK and SP-MSD. HCR does not own any stocks, nor has other interests in these companies. PDG is employed by the commercial entity that sponsored the study. SF is employed by the commercial entity that sponsored the study.

## Authors' contributions

All authors were members of the project team for this study. Their roles are specified below.

JHWvdB designed the study and wrote the manuscript. AMS performed the data-analysis and commented on all drafts of the manuscript. HCR commented on the internet survey questionnaire, and on all drafts of the manuscript. PDG commented on the internet survey questionnaire, and on all drafts of the manuscript. SCF commented on all drafts of the manuscript. All authors read and approved the final manuscript.

## Pre-publication history

The pre-publication history for this paper can be accessed here:

http://www.biomedcentral.com/1471-2334/11/320/prepub
